# Can ongoing movements be guided by allocentric visual information when the target is visible?

**DOI:** 10.1167/jov.21.1.6

**Published:** 2021-01-11

**Authors:** Emily M. Crowe, Martin Bossard, Eli Brenner

**Affiliations:** 1 Department of Human Movement Sciences, Institute of Brain and Behaviour Amsterdam, Amsterdam Movement Sciences, Vrije Universiteit Amsterdam, Amsterdam, The Netherlands; 2 School of Psychology, Cardiff University, Cardiff, UK

**Keywords:** online, arm movements, interception, perturbation, egocentric

## Abstract

People use both egocentric (object-to-self) and allocentric (object-to-object) spatial information to interact with the world. Evidence for allocentric information guiding ongoing actions stems from studies in which people reached to where targets had previously been seen while other objects were moved. Since egocentric position judgments might fade or change when the target is removed, we sought for conditions in which people might benefit from relying on allocentric information when the target remains visible. We used a task that required participants to intercept targets that moved across a screen using a cursor that represented their finger but that moved by a different amount in a different plane. During each attempt, we perturbed the target, cursor, or background individually or all three simultaneously such that their relative positions did not change and there was no need to adjust the ongoing movement. An obvious way to avoid responding to such simultaneous perturbations is by relying on allocentric information. Relying on egocentric information would give a response that resembles the combined responses to the three isolated perturbations. The hand responded in accordance with the responses to the isolated perturbations despite the differences between how the finger and cursor moved. This response remained when the simultaneous perturbation was repeated many times, suggesting that participants hardly relied upon allocentric spatial information to control their ongoing visually guided actions.

## Introduction

In order to successfully interact with the world, we must encode, organize, and use spatial information. Such information can be represented in two fundamentally different ways. Egocentric information is defined relative to the self, whereas allocentric information describes object-to-object relations independent of the self. This distinction is believed to be critical for the way in which the visual system is organized. A ventral visual pathway that is primarily involved in tasks that require persistent relationships, such as recognizing people or objects, is proposed to organize visual input allocentrically, while a dorsal visual pathway primarily guides ongoing actions using instantaneous egocentric spatial information (Goodale & Milner, 1992).

Although the distinction is often referred to as one between perception and action, not all actions have a straightforward place within this scheme. Memory-guided actions, for instance, are movements in which a target object is removed from view prior to a motor response. Such movements are guided by remembered target positions, so they presumably depend on the ventral system that stores persistent information to some extent (Goodale, 2008). This contrasts with actions toward visible targets that might rely exclusively on the dorsal system in which ongoing movements are updated online according to moment-to-moment information about the location of a target object. Westwood and Goodale (2003) proposed that the kind of information that is used depends on when the target is visible, suggesting that the target does not need to be visible throughout the entire movement for the action to be guided by dorsal, egocentric information. Moreover, evidence is accumulating against a strict separation between the two pathways in terms of goals and representations (Schenk & McIntosh, 2010). Thus, it is not inconceivable that allocentric information could guide ongoing movements under certain circumstances.

Lu and Fiehler (2020) recently adapted their well-established paradigm (e.g., Fiehler, Wolf, Klinghammer, & Blohm, 2014; Klinghammer, Blohm, & Fiehler, 2015, 2017) by administering an air-puff to the right eye of participants to force an eye-blink. During the blink, the target item disappeared, and on the critical trials, other objects (i.e., landmarks) were shifted. The puffs were presented at various times with respect to movement onset and were classified as memory guided, memory guided delayed, or online according to Westwood and Goodale's (2003) real-time hypothesis. Irrespective of the time of the eye-blink, and therefore of the object displacements, reaching movements were corrected in accordance with the updated location of the landmarks, indicating the use of allocentric information. The authors interpret this result as the first evidence for the use of allocentric information in real-time reaching. A limitation of Lu and Fiehler's (2020) work is that, after the eye-blink, there was no visual information regarding the target because the target was removed from the test scene. This is, of course, a requirement of the experimental paradigm: The target had to be removed to allow for a modification of allocentric but not egocentric information, which was necessary to be able to determine the weighting of these two types of spatial information. To search for evidence for the use of allocentric information in an ongoing visually guided action when the target remains visible, we sought for a task in which it could be advantageous for participants to rely on allocentric information despite the target being visible.

It is generally accepted that using a tool changes the relationship between the “self” and the “surroundings” to some extent (see Holmes, 2012, for a review). Several *types* of tools exist. Some tools, when gripped by our hand, can be considered extensions of that hand. This is, for instance, the case when using a stick to intercept a ball drifting along a canal. In this example, we get tactile feedback from the stick and watch our arm perform a movement that logically leads to the interception. For other tools, the relationship between the task and how the hand moves is less straightforward. This is, for instance, so when turning the steering wheel of a car or using a cursor to intercept a virtual ball drifting along a virtual canal on a computer screen. In the latter example, we do not get tactile information regarding the interception and cannot directly perceive the relationship between how our arm moves and how the cursor moves to intercept the ball. Actually, when moving a cursor, the hand normally moves forward to move the cursor upward on the screen, and the extents of the hand and cursor movements can be quite different. It is not even obvious where the origin of the egocentric reference would be when moving a cursor across a screen. It is therefore reasonable to assume that such a tool increases the extent to which one relies upon allocentric visual information to guide the action. We therefore used such a tool to examine whether *ongoing movements* can be guided by allocentric spatial information.

Perturbation paradigms have commonly been used to explore how sudden unpredictable changes in the visual input influence goal-directed movements. The hand has been reported to deviate from its path in the direction of a target perturbation (e.g., Georgopoulos, Kalaska, & Massey, 1981; Franklin, Reichenbach, Franklin, & Diedrichsen, 2016; Reichenbach, Franklin, Zatka-Haas, & Diedrichsen, 2014; Soechting & Lacquaniti, 1983). This response occurs approximately 100–150 ms after the perturbation (Brenner & Smeets, 1997; Day & Lyon, 2000; Gritsenko, Yakovenko, & Kalaska, 2009; Oostwoud Wijdenes, Brenner, & Smeets, 2011 ; Prablanc & Martin, 1992). Research has also shown that the hand deviates in the direction opposite to a cursor perturbation with a similar latency (Brenner & Smeets, 2003; Brière & Proteau, 2010; Cross, Cluff, Takei, & Scott, 2019; Franklin et al., 2016; Proteau, Roujoula, & Messier, 2009; Reichenbach et al., 2014; Sarlegna et al., 2004; Sarlegna & Blouin, 2010; Saunders & Knill, 2004; Veyrat-Masson, Brière, & Proteau, 2010). Importantly for the current study, the hand has also been reported to deviate in the direction of sudden unexpected background motion with a latency of approximately 110–160 ms (Gomi, Abekawa, & Nishida, 2006; Gomi, Abekawa, & Shimojo, 2013; Saijo, Murakami, Nishida, & Gomi, 2005; Whitney, Westwood, & Goodale, 2003) even when the target remains visible (Brenner & Smeets, 1997).

The existing literature shows that corrective responses to target, cursor, and background perturbations are robust and occur with a similar latency. This study aimed to explore whether moving a cursor relies upon allocentric information by applying perturbations to these different components (i.e., target, cursor, background) of an interception task either independently or simultaneously. When all the components of the task move simultaneously, the spatial relations between the target, cursor, and background remain constant, whereas the spatial relations between the observer and the task components change. Therefore, if only allocentric information is used to guide the cursor in this task, we would not expect any corrective responses to the simultaneous perturbation of all three task components. In a first experiment, we examined whether participants responded to such simultaneous perturbation and, if so, whether responses were consistent with the responses to the separate components. In a second experiment, we examined whether participants would learn not to respond to simultaneous perturbations if such perturbations were presented repeatedly.

## Method

### Participants

Twelve students (nine females; 11 right-handed; 21.3 ± 4.5 years of age, mean ± standard deviation) from the Vrije Universiteit Amsterdam participated in return for course credit. Participants were unaware of the purpose of the study. The study was approved by the Ethics Committee of the Vrije Universiteit Amsterdam in accordance with the Declaration of Helsinki. All participants gave written informed consent and were debriefed at the end of the experiment.

### Experimental setup

**Figure 1.**
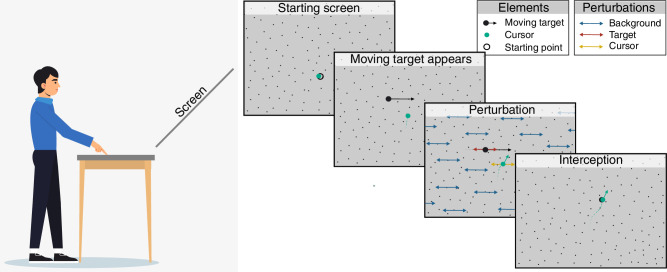
Schematic representation of the task. Participant stood behind a table and used their dominant index finger, which was represented by a green disc (cursor) on the screen, to intercept a black moving target. To start a trial, participants had to place and keep the cursor at the starting point until the moving target appeared. The target moved rightward across the screen. A perturbation lasting 100 ms was applied to the target, cursor, or background or to all three simultaneously, 300 ms after the moving target appeared. Participants’ task was to intercept the moving target using the green cursor.

The experiment was conducted in a normally illuminated room. The stimuli were back-projected at 120 Hz with a resolution of 800 × 600 pixels onto a 1.20 × 1.00-m acrylic rear-projection screen (Techplex 15, Stewart Filmscreen Corporation, Torrance, California, USA) tilted backward by 30⁰. Participants stood approximately 1 m from the screen. There was a table between them and the screen. To complete the task, participants moved their dominant index finger along the surface of this table. The position of their finger was represented by a green cursor presented on the screen (Figure 1).

An infrared camera system (Optotrak 3020; Northern Digital, Waterloo, ON, Canada) recording at 500 Hz was placed approximately at shoulder height above the ground to the left of the screen. A marker (an infrared light-emitting diode) was attached to the nail of the participants’ dominant index finger. In order to synchronize the movement data (the measured marker positions) with the stimulus presentation, the camera also recorded the position of a second marker attached to the side of the screen. This marker did not move, but it stopped emitting infrared light so that its position was registered as “missing” when a flash was presented at the top-left corner of the screen (where a light sensor was placed to detect the flash).

### Calibration

At the beginning of each session, the position of the marker on the fingertip was measured when the fingertip was at four indicated positions on a sheet of paper that was placed at a fixed position on the table. The four positions were at the corners of an invisible rectangle with sides of 21 and 15 cm. This simple four-point calibration was used to relate the position of the fingertip to that of the cursor in the projected images, whereby the four calibrated points coincided with the centers of the four quadrants of the screen. The sheet of paper was removed after the calibration.

### Stimulus and procedure

Throughout the experiment, the participants’ hand moved across the surface of the table in front of them and the position of their dominant index finger was represented by a green cursor on the screen (see Figure 1). The background was a gray surface with 600 randomly placed black dots of 1 cm diameter. The target was a 6-cm diameter black disk. The starting point was a 4-cm diameter black disk that was 10 cm below the screen center. Participants started a trial by placing the cursor on the starting point and keeping the cursor there until the target appeared. If they moved the cursor before the target appeared, the target did not appear and they had to move the cursor back to the starting point. Between 500 and 700 ms after the cursor reached the starting point, the starting point was removed and the target appeared 20 cm to the left of and 10 cm above the screen center. The target moved to the right at 30 cm/s. The perturbations always took place 300 ms after the target appeared and always consisted of 20 cm/s (additional) motion to the left or to the right that lasted for 100 ms. Thus, the perturbation always gave rise to a 2-cm displacement on the screen with respect to where each task component would have been without the perturbation. There were four possible kinds of perturbations:

1) For target perturbations, the target velocity either increased to 50 cm/s (for a rightward perturbation) or decreased to 10 cm/s (for a leftward perturbation).
2) For background perturbations, the background dots all moved at 20 cm/s for 100 ms. Dots that moved off the side of the screen were replaced by new dots at the other side.
3) For cursor perturbations, the cursor position was displaced from the calibrated position corresponding to the hand's position at 20 cm/s. Consequently, it ended up being 2 cm further to the left or right of the position defined by the calibrated relationship between finger and cursor. Note that it was the cursor that underwent the 2-cm displacement. The finger only needed to move 0.7 cm to compensate for this.
4) For simultaneous perturbations, the target, cursor, and background all moved in the manner described above, either all to the left or all to the right.

The participants’ task was to *intercept* the target by sliding their finger through it at any location along its path (i.e., there was no predefined interception zone). When the cursor crossed the target's path, whether it had hit the target was determined by examining whether the linearly interpolated position of the center of the cursor was ever within the interpolated bounds of the target. If participants were successful, the target disappeared and participants heard a sound. If they were unsuccessful, the target continued moving along its trajectory until it disappeared. The current trial number was presented in the bottom-left corner of the screen, and participants could rest whenever they wanted to by not bringing the cursor to the starting point. Participants could stand and move in any way they felt would help them perform the task, so all measurements are presented in centimeters rather than degrees of visual angle, because the latter differed between participants and trials.

### Experiment 1

In Experiment 1, the four kinds of perturbations were randomly interleaved. For each of these four kinds of perturbations, participants completed 50 trials with added leftward motion and 50 trials with added rightward motion. The total of 400 interleaved trials took approximately 20 min. The purpose of this experiment was to determine whether participants used allocentric spatial information to visually guide the ongoing movement of a cursor on a screen.

### Experiment 2

In Experiment 2, there was only one kind of perturbation: The target, cursor, and background were always perturbed simultaneously. Participants completed 200 trials with added leftward motion and 200 trials with added rightward motion. These 400 interleaved trials also took approximately 20 min. The purpose was to determine whether people would learn to rely more on allocentric information, ignoring overall shifts of the whole scene, if such overall shifts occurred on every trial.

### Analysis

Finger coordinates were measured at 500 Hz. To evaluate the time course of responses to the perturbations, we analyzed the measured horizontal positions of the finger for 290 ms from the time of the perturbation. This time period was considered adequate because we expected responses to start approximately 150 ms after the perturbation (Brenner & Smeets, 2015). We did not consider later moments because we did not want to include trials in which movements had crossed the target's path. Choosing to analyze positions until 290 ms after the perturbation meant that individual trials were excluded if the target was intercepted less than 590 ms after it appeared. Trials were also excluded if there were position changes of 50 cm or more between consecutive samples (which must be due to measurement errors, possibly occasionally detecting reflections of the marker rather than the marker itself). With these choices, in total, 858 of the 9,600 trials were removed (8.9%).

**Figure 2.**
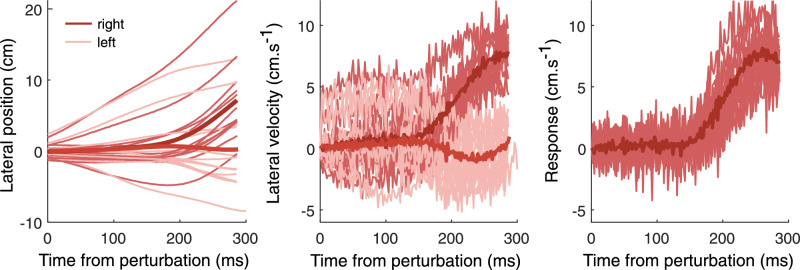
Example of the steps in the data analysis. Response of the finger to the target perturbation in Experiment 1. Positive values for the lateral position and velocity are to the right. A positive response is in the direction of the perturbation. Thin lines show mean values for individual participants. Thick lines show means across participants. Left panel: Lateral position of the finger as a function of the time that has passed since the onset of the perturbation. Each participant is represented by two lines, one for a perturbation to the right and one for a perturbation to the left. Middle panel: The same data presented as the lateral velocity of the finger as a function of time. Right panel: Difference between the lateral velocities after rightward and leftward target perturbations.

For each participant, we calculated the average finger position for every 2-ms time interval from the moment of the perturbation (see example in left panel of Figure 2). In Experiment 1, we did so separately for both types of added motion (i.e., leftward, rightward) and for each of the four kinds of perturbation. In Experiment 2, we did so separately for the first, second, third, and final set of 50 trials for both types of added motion. These average positions were then converted into velocities (central panel of Figure 2), from which responses were calculated for each kind of perturbation or set of trials by subtracting the average horizontal velocity for added leftward motion from the average horizontal velocity for the corresponding added rightward motion (right panel of Figure 2). A positive response indicates that the hand followed the direction of the perturbation. A negative response indicates that the hand moved in the opposite direction. These values were then averaged across the 12 participants.

The left panel of Figure 2 shows that there was quite a lot of variability between the different participants’ hand movements. In particular, two participants moved further to the right and one participant moved further to the left than most others. Such variability across participants is not surprising, because we did not instruct participants where (or when) to intercept the target. Obviously, if they moved in a certain manner, they did so irrespective of the perturbation (until they responded to the perturbation; note how the traces clearly come in pairs for the three participants mentioned above), so such differences disappear when the response is determined by subtracting the lateral velocity after leftward perturbations from the lateral velocity after rightward perturbations. The freedom to choose where to intercept the target may have introduced some variability in the vigor of the response (Oostwoud Wijdenes, Brenner, & Smeets, 2011), but we were not worried about this because each participant provided data for all conditions. Similarly, to reduce the required number of trials, we did not include trials without a perturbation but directly compared the responses to leftward and rightward perturbations. The responses to leftward and rightward perturbations are probably symmetric, as they have previously been found to be for the target (Franklin et al., 2016), cursor (Franklin et al., 2016), and background (Brenner & Smeets, 2015), but such symmetry is not required for our analysis.

We quantified the initial response to the perturbations by taking the mean response between 150 and 250 ms after the onset of the perturbation. We expected the responses to all the perturbations to have started by 150 ms after their onset (Brenner & Smeets, 2003) and only considered 100 ms from that time because, after that, responses are likely to be influenced by visual feedback about the adequacy of the responses. We determined the initial response for each participant and used these values for our statistical analysis. In Experiment 1, we assessed whether there was a consistent response in the simultaneous perturbation condition with a one-sample *t* test. Evidence of a response in this condition would suggest that an egocentric reference frame was used. To evaluate whether the individual participants’ responses are what one would expect on the basis of their responses to the three isolated perturbations on their own, we determined the correlation between their responses in the simultaneous perturbation condition and the sum of their responses in the other three isolated perturbation conditions. In Experiment 2, we used a one-sample *t* test to assess whether a response was still present during the final 100 trials, after participants had been exposed to 300 trials of the simultaneous condition, the condition for which it might be advantageous to rely on an allocentric reference frame.

## Results

### Experiment 1

**Figure 3.**
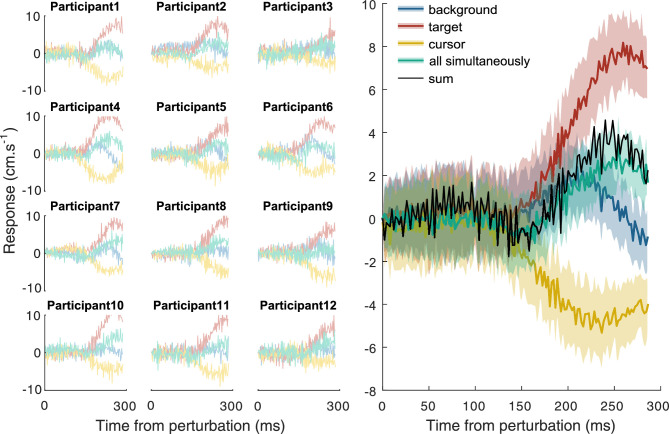
Each curve shows the difference between the mean lateral velocity of the finger after rightward and leftward perturbations, either for individual participants (left panel) or averaged across participants (right panel). A positive response is in the direction of the perturbation, and a negative response is in the direction opposite to the perturbation. Shaded regions in the right panel show the standard error across participants. Colors indicate the kind of perturbation. The thin black curve shows the sum of the average responses to the separate background, target, and cursor perturbations. “All simultaneously” refers to the background, target, and cursor moving together.

Participants intercepted the target on 96% ± 4%, 96% ± 3%, 96% ± 5%, and 94% ± 4% of the trials (mean ± standard deviation) when the background, target, cursor, or all three were perturbed. Thus, participants corrected adequately for both the target and cursor perturbations. Figure 3 shows that participants responded to all types of perturbation approximately 150 ms after the onset of the perturbation. As expected, participants’ fingers followed the direction of the background and target perturbation (positive responses) and moved in the opposite direction to the cursor perturbation (negative response). A one-sample *t* test showed that when all the components moved simultaneously, there was a response of the hand in the direction of the perturbation, *t*(11) = 4.92, *p* < 0.001. This response was almost identical to the response that one would predict from the way in which the participants responded to the three individual components (black line). Individual differences between the magnitudes of such responses were correlated with such predictions for the same participants (*r* = .653*, p* = 0.021).

### Experiment 2

**Figure 4.**
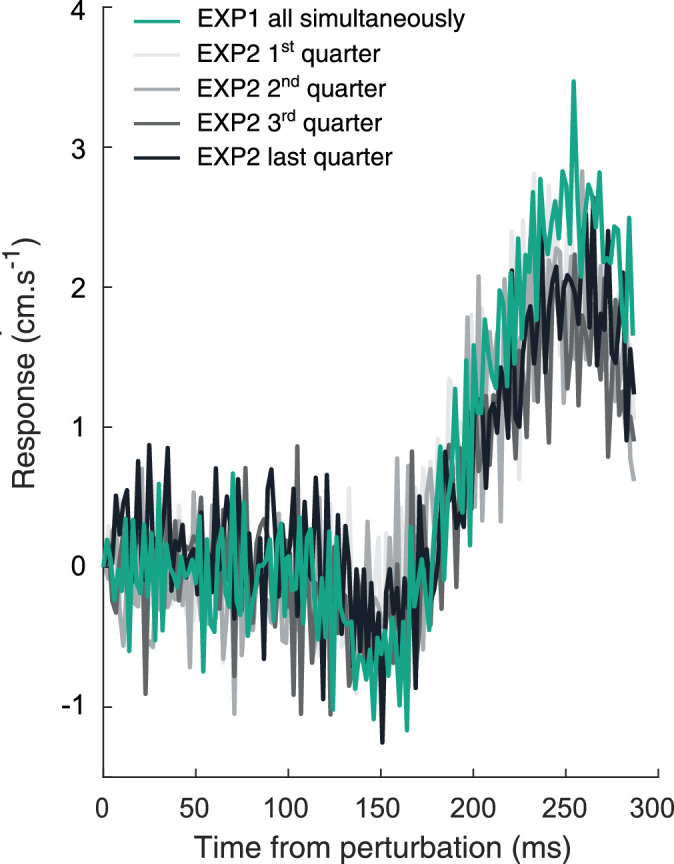
Each curve shows the difference between the lateral velocity of the finger after rightward and leftward perturbations, averaged across participants. A positive response is in the direction of the perturbation. The different colors show participants’ responses at different times during the experiment, with the different shades of gray representing consecutive sets of 50 trials. The green line shows the value for the same (all simultaneously) perturbation in the first experiment.

Participants intercepted the target on 98% ± 2%, 98% ± 2%, 99% ± 2%, and 98% ± 2% of the trials during the first, second, third, and last quarters of the trials. As in Experiment 1, participants responded to the simultaneous perturbation of the target, background, and cursor in the direction of the perturbation (Figure 4). This occurred approximately 150 ms after the onset of the perturbation. Most importantly, the hand continued to respond throughout the experiment, although the relative positions of the target, cursor, and background were never perturbed. The response was still evidently present in the last quarter of the experiment (black curve in Figure 4; *t*(11) = 2.61, *p* = 0.024). Indeed, the response in the final quarter was very similar to the response in the earlier quarters (gray curves) and also very similar to the response to interleaved trials of the same simultaneous perturbation in Experiment 1 (reproduced here as the green curve).

## Discussion

Participants were instructed to intercept a moving target with a cursor that they controlled by moving their finger across the surface of a table in front of them. In Experiment 1, we examined how participants responded to perturbations of the target, cursor, background, and all three simultaneously. When all three components of the task moved simultaneously, there was a change in egocentric but not allocentric visual information (unless one considers objects beyond the screen). Since participants responded to the simultaneous perturbations, ongoing movements were clearly not guided exclusively by information about the relative positions of the target, cursor, and background (i.e., by allocentric information). The response to simultaneous perturbations of all three components coincided quite accurately with the sum of the responses to shifts of each components on its own (Experiment 1), as one might expect if perturbations of each of the (egocentric) positions were dealt with separately. The response even started with a modest shift in the opposite direction than the perturbation, in accordance with the response to perturbing the cursor appearing to be faster than that to perturbing the target under these circumstances. In Experiment 2, simultaneous perturbations were presented on every trial to explore whether participants were more likely to rely on allocentric information when it was advantageous to do so because this would reveal that there was no need to respond at all. Participants’ responses were very similar to those in Experiment 1. This suggests that even in circumstances in which one might expect people to rely heavily on allocentric information, they do not do so.

Previous research has shown that a combination of egocentric and allocentric information can be used to guide movements toward remembered positions and that the weighting of such information is modulated by various factors (e.g., Byrne et al., 2010; Klinghammer et al., 2017). Our results obviously cannot show that allocentric information is never used or even that it is not used at all under the current circumstances. What it does show is that relative positions play a rather minor role. We found similar average responses when all the task components moved simultaneously and when we summed the responses when each task component was perturbed in isolation. We also found a significant correlation between these two measures of individual participants’ responses. This suggests that the individual participants dealt with each perturbation in the same way when it was presented alone as when they were all presented together, which one would not expect if participants were responding to relative motion (i.e., allocentric information). This was the case, although the circumstances were such that one might expect the role of allocentric information to be larger than usual. We assume that egocentric information is easier to rely on when it is an actual part of one's body that is moving to the target, such that it is evident how one should adjust the movement in response to errors that are observed. The assumption that the use of egocentric information relies on an anatomical link between where we observe things and the posture that is required to reach them might be incorrect. Our results suggest that moving a cursor to a target by moving one's finger in a different plane can be guided by egocentric information. This can be done despite having to consider the differences in the direction and magnitude of motion between finger and cursor, in line with a recent study showing that participants had a good intuition of how to use a computer mouse when the mapping between one's hand and the cursor was close to what they are used to (Brenner et al., 2020). We have no idea how this is achieved, but apparently guiding the cursor to the target by moving one's finger elsewhere is possible without resorting to relying exclusively on allocentric information.

The current experiment was completed in a normally illuminated room such that surrounding stationary items, including the edges of the screen, could have been used as a reference. For target and cursor perturbations, it is irrelevant whether the static background or other static items are used as the reference, but for background perturbations, it is obviously not. The fact that background perturbations alone did influence the movements suggests that the background is used as a reference, possibly as part of a mechanism to compensate for self-motion (Brenner & Smeets, 1997; Gomi, 2008). We expected to find a response to background perturbation, despite surrounding static structures being visible, because the effect of background motion is known to mainly rely on the regions close to where the finger is going (Brenner & Smeets, 2015) and where gaze is directed (Abekawa & Gomi, 2010). Similarly, studies that demonstrated that allocentric information helps guide the hand to remembered positions have shown that only certain surrounding items are considered (Fiehler et al., 2014; Lu & Fiehler, 2020). This does not exclude the possibility that surrounding stationary items play a modest role as a reference and that the response to background motion would have been larger if the surrounding were not visible. However, whatever influence the background perturbation has as a reference does not change when the other two items, the target and cursor, move together with it. Thus, as long as we accept that the influence of moving the background is a result of it being used as a reference (i.e., that the position of the observer or of the target and cursor is in some way judged relative to the background), our conclusion is justified.

Previous research has shown that allocentric information can play an important role in guiding movements (e.g., Fiehler, Wolf, Klinghammer, & Blohm, 2014; Karimpur, Eftekharifar, Troje, & Fiehler, 2020; Lu & Fiehler, 2020). An important difference between such research and the present study is that participants could see both the target and the cursor throughout the entire movement in our experiments. Apparently, when the most relevant items remain visible throughout the movement, participants primarily rely on judgments of the items’ egocentric positions, even under circumstances in which relying on allocentric spatial information could be advantageous because doing so would reveal that the relative positions of the relevant items on the screen did not change. We propose that the clear role of allocentric information in reaching that was found in studies using dynamic landmark information (Lu & Fiehler, 2020) is related to the uncertainty about the target location as a result of not being able to see the target, which could bias participants to use an allocentric reference frame (Byrne & Crawford, 2010). Even if the dorsal visual pathway relies exclusively on instantaneous egocentric information and is responsible for quickly guiding the hand to the target, one might expect allocentric information to start playing a substantial role when instantaneous egocentric information about the target is removed, so that one has to rely on memory. When encoding spatial locations, one is known to partially rely on landmarks (e.g., Carrozzo, Stratta, McIntyre, & Lacquaniti, 2002; Krigolson & Heath, 2004; Schütz, Henriques, & Fiehler, 2013). Therefore, even if actions are guided by egocentric information, when the target is not visible, its egocentric position is presumably judged from the scene in a manner that involves allocentric information, namely, the targets’ position relative to the remaining visible items.

## Conclusions

We present two experiments that investigated whether ongoing movements can be guided by allocentric visual information when the target remains visible throughout the entire movement. In the task we selected, it would be advantageous for participants to use allocentric information to avoid making unnecessary adjustments during an ongoing movement. However, in both experiments, participants responded to the simultaneous perturbation of the target, cursor, and background, indicating that allocentric spatial information alone could not be used. The extent to which they did so suggests that they hardly relied on allocentric information, if at all. The results also show that details of a task such as whether the target remains visible can influence the extent to which allocentric information is used.
